# Tailoring Optical Forces Behavior in Nano-optomechanical Devices Immersed in Fluid Media

**DOI:** 10.1038/s41598-017-14777-z

**Published:** 2017-10-30

**Authors:** Janderson R. Rodrigues, Vilson R. Almeida

**Affiliations:** 1 Instituto Tecnológico de Aeronáutica, São José dos Campos, SP, 12228-900 Brazil; 2 Instituto de Estudos Avançados, São José dos Campos, SP, 12228-001 Brazil

## Abstract

Emerging nano-optofluidic devices have allowed a synergetic relation between photonic integrated circuits and microfluidics, allowing manipulation and transport at the realm of nanoscale science. Simultaneously, optical gradient forces have allowed highly precise control of mechanical motion in nano-optomechanical devices. In this report, we show that the repulsive optical forces of the antisymmetric eigenmodes in an optomechanical device, based on a slot-waveguide structure, increases as the refraction index of the fluid medium increases. This effect provides a feasible way to tailor the repulsive optical forces when these nano-optomechanical devices are immersed in dielectric liquids. Furthermore, the total control of the attractive and repulsive optical forces inside liquids may be applied to design novel nanophotonic devices, containing both microfluidic and nanomechanical functionalities, which may find useful applications in several areas, such as biomedical sensors, manipulators and sorters, amongst others.

## Introduction

Advances in nanosciences and nanotechnologies have emphasized the need for new manipulation and transport techniques at nanoscale. The precise and stable manipulation and transport of nanoparticles, nanoclusters, or molecules is a critical step to advance these areas. In order to overcome these challenges, a wide variety of methods have been proposed; amongst these, the light-matter interaction methods have been drawing attention as, for instance, optofluidic devices^1,2^. These devices have merged, in a synergic manner, photonic integrated circuits (PICs) with optofluidic transport, for chemistry and biological applications^3^. Recently, optofluidic devices based on silicon slot waveguides immersed in water have been successfully applied for manipulating and transporting dielectric nanoparticles and DNA molecules using infrared light^4–6^.

On the other hand, optical gradient forces have allowed for the control of nanomechanical dielectric devices^7,8^. These optical forces originate from electric dipoles moments induced in the dielectric materials by the light intensity gradient of spatial distributions of the guided modes. Optical gradient forces may be either attractive or repulsive, depending on the resulting dipoles phases, which are dictated by the optical and geometrical properties of the structures^9^. In a dielectric planar (slab) slot waveguide, the symmetric modes lead to attractive forces, whereas the antisymmetric ones lead to repulsive forces^10,11^. However, in a realistic rectangular cross-section slot waveguide, the optical gradient forces for symmetric modes are still attractive, but for antisymmetric modes they switch from attractive (for narrow gaps) to repulsive (for wide gaps). Therefore, there is a cross-over gap, after what the repulsive optical force emerges^9^. In this report, we show that it is possible to decrease and even eliminate the cross-over gap, by immersing the nanowaveguide structures in appropriate dielectric fluid media, thus making it possible to tailor the repulsive optical forces.

## Results and Discussions

**Figure 1:**
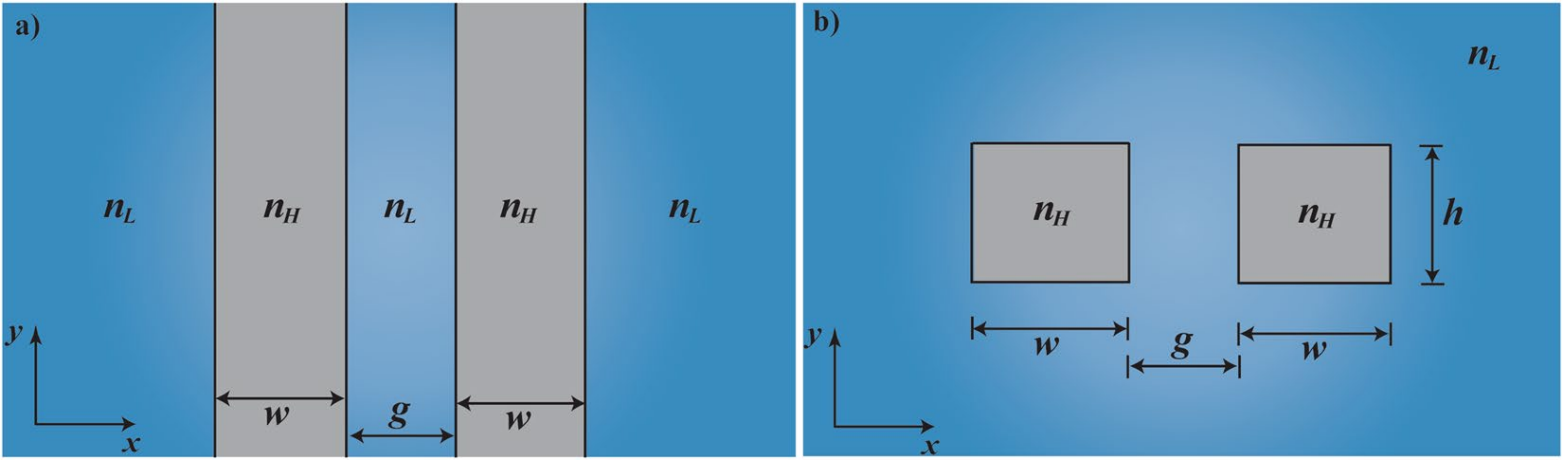
Schematic illustrations of the dielectric planar and rectangular cross-section slot waveguides. Dielectric slot-waveguide structures composed of two symmetric high-index waveguides with refractive index *n*_*H*_, immersed in a low-index fluid medium with refractive index *n*_*L*_. **(a)** Planar slot waveguide. **(b)** Rectangular slot waveguide.

We consider the slot waveguide structure, shown schematically in Fig. 1, which is composed by two high-index (*n*_*H*_) coupled waveguides, separated by a gap g, immersed in a low-index dielectric medium (*n*_*L*_). For gap separations much shorter than the decay of the evanescent fields of the individual waveguides, the slot waveguide provides enhancement and high confinement of light inside the gap region, known as slot effect^12,13^. Due to the geometric simplicity of the planar slot structure, shown in Fig. 1(a), the optical forces can be carried out analytically by using Maxwell’s equations, Poynting vector concept, and Stress Tensor formalism^10,11^. Alternatively, the optical forces *F*_*opt*_ per unity area (length in *z*-direction and height in *y*-direction) for the planar structure can be obtained by the following dispersion relation^11^:

1 $$\frac{{F}_{opt}({\rm{g}})}{hL}=\frac{1}{c}\frac{d{n}_{eff}({\rm{g}})}{d{\rm{g}}}\frac{P}{h},$$

where *n*_*eff*_ is the eigenmode effective index, *P/h* is optical power per unit high in y-direction, and *c* is the velocity of light in vacuum. On the other hand, the optical force in the rectangular structure shown in Fig. 1(b), is given very similarly by^14,15^:

2 $$\frac{{F}_{opt}({\rm{g}})}{L}=\frac{1}{c}\frac{d{n}_{eff}({\rm{g}})}{d{\rm{g}}}P.$$

### Planar slot waveguide analysis

**Figure 2:**
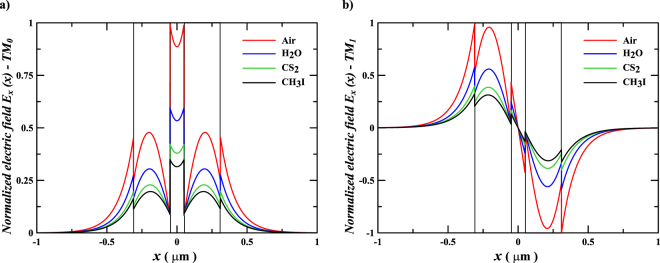
Electric field distributions in the planar slot waveguide. Spatial distributions of the normalized *E*_*x*_(*x*) for TM polarization of a silicon planar slot waveguide with g = 100 nm, immersed in Air, H_2_O, CS_2_, and CH_3_I, at a wavelength of *λ*_0_ = 1550 nm. (**a**) Symmetric TM mode – TM_0_. **(b)** Antisymmetric TM mode – TM_1_.

We analyze silicon (*n*_*H*_ = 3.4764)^16^ slot waveguide structures with four different dielectric fluid claddings: air – Air (*n*_*L*_ = 1.0003)^17^, water – H_2_O (*n*_*L*_ = 1.3154)^18^, carbon disulfide – CS_2_ (*n*_*L*_ = 1.5884)^18^, and modified methyl iodide – CH_3_I (*n*_*L*_ = 1.7630)^19^, at a light source wavelength of *λ*_0_ = 1550 nm. The normalized *x*-component of the electric field spatial distributions, *E*_*x*_(*x*), for TM polarization, are shown in Fig. 2(a) for the fundamental symmetric mode TM_0_, and in Fig. 2(b) for the antisymmetric mode TM_1_, for a planar slot waveguide structure with width *w* = 310 nm and gap g =100 nm. The results show that the light intensity for the TM_0_ mode is highly confined in the gap region, due to the discontinuities of the transverse component of the electric at dielectric interfaces – the slot effect^12^. The amplitude ratio between the electric field inside the gap and that at the waveguides’ interfaces is given by ${{n}_{H}}^{2}/{{n}_{L}}^{2}$; therefore, this discontinuity decreases as the fluid medium index increases. The transverse discontinuity of the electric field for the TM_1_ mode also decreases as the fluid medium index increases.

**Figure 3:**
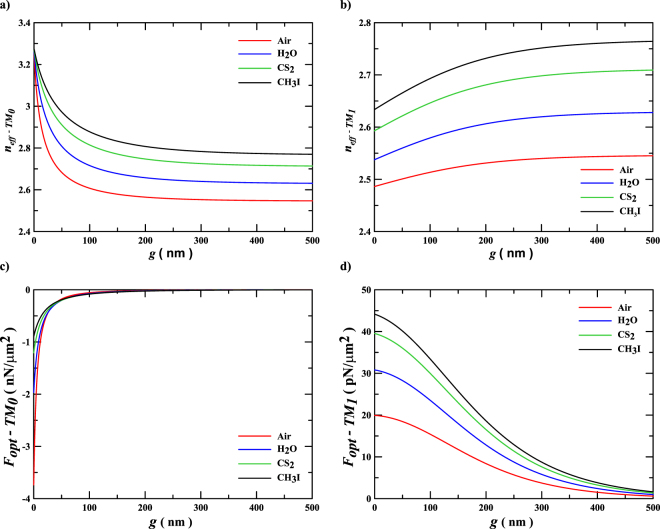
Effective indexes and optical forces of the dielectric planar slot waveguide. Effective indexes (**a,b**) and optical forces (**c,d**) of the silicon planar slot structure, with width *w* = 310 nm for an optical power per unit length of *P*/*h* = 20 mW/*μm* at wavelength *λ*_0_ = 1550 nm, immersed in various dielectric media, as a function of the gap distance for the fundamental symmetric mode TM_0_ (**a,c**) and the antisymmetric mode TM_1_ **(b**,**d)**.

The effective index, *n*_*eff*_, and the optical forces, *F*_*opt*_, for the silicon planar slot waveguide, as a function of the gap g are presented in Fig. 3. Figure 3(a and b) show that the effective indexes of both modes increase as the fluid medium refractive index increases. However, the effective index of the TM_0_ mode decreases as the gap increases, whereas the effective index of the TM_1_ mode increases. Furthermore, the effective indexes converge asymptotically to specific values as the gap increases to large distances, corresponding to the effective indexes of the individual waveguides in a decoupled system. Figure 3(c and d) show the optical forces for the TM_0_ and TM_1_ modes, respectively, for an optical power per unit length of P/h = 20 mW/*µm*. In planar slot waveguides, the symmetric modes result in attractive (negative) optical forces, whereas the antisymmetric ones result in repulsive (positive) optical forces^10,11^. For very narrow gaps, neglecting Casimir and van der Waals (dispersions) forces^20^, the maximum absolute optical force is achieved by the TM_0_ mode, when the structure is embedded in Air (−3.75 nN/*µm* ^2^), due to the enhanced slot effect in this case, and it decreases as the fluid medium refractive index increases, reaching the lowest comparative magnitude (−0.9 nN/*µm* ^2^) for CH_3_I, as shown in Fig. 3(c). On the other hand, the repulsive optical force of the TM_1_ increases as the medium index increases. The repulsive optical force reaches +20 pN/*µm* ^2^ for Air as fluid medium, and it increases to +44 pN/*µm* ^2^ for CH_3_I. This unique behavior cannot be understood neither by the dispersion relation in the Eq. (1) nor by the field distribution shown in Fig. 2(b); however, analyzing the stress tensor version of the optical force, presented in Eq. (1), which shows that, for the TM polarization, the effective index of the antisymmetric mode has a dominant dependence factor, almost proportional to ${{n}_{L}}^{4}$ (see Appendix A).

### Rectangular slot waveguide analysis

**Figure 4:**
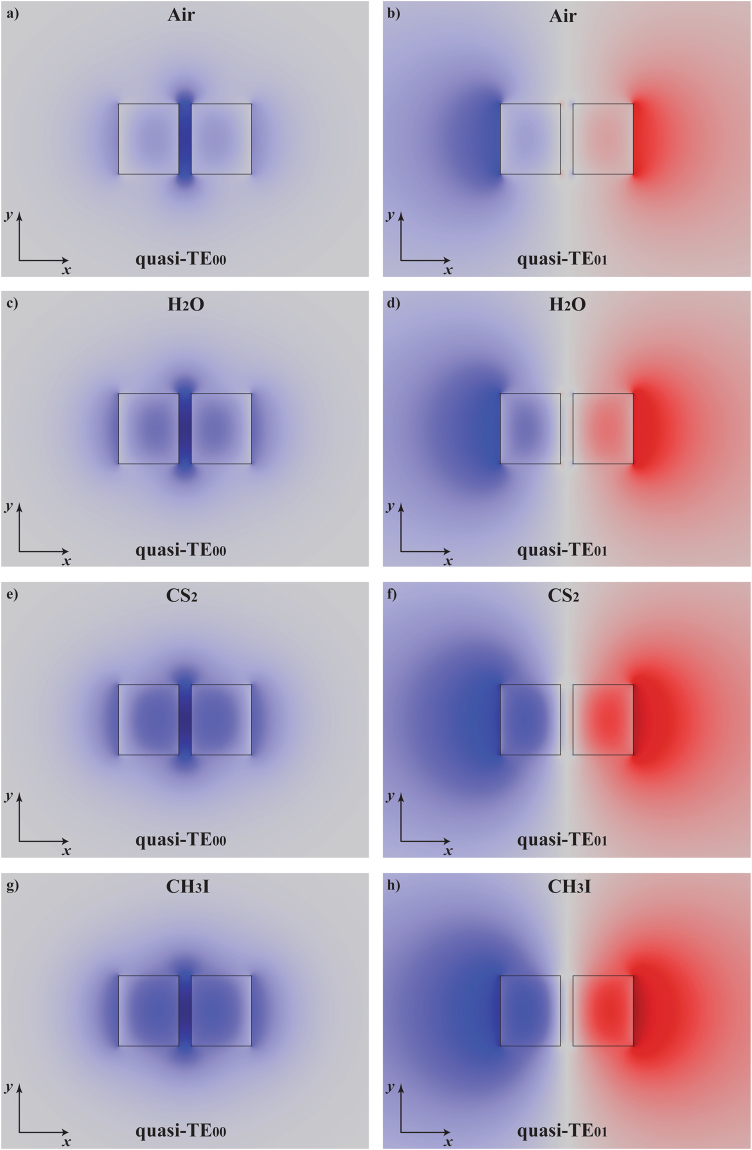
Electric field distributions in the rectangular slot waveguide. FEM eigenmode simulations showing *E*_*x*_(*x*, *y*) for the symmetric – quasi-TE_00_ (left column) and antisymmetric – quasi-TE_01_ (right column) modes; *λ*_0_ = 1550 nm, *w* = 280 nm, *h* = 240 nm, and g = 50 nm; slot waveguide immersed in Air (**a**,**b**), H_2_O (**c,d**), CS_2_ (**e**,**f**), and CH_3_I (**g**,**h**).

In contrast with the planar structure, the more realistic rectangular cross-section structure presents finite-dimension restriction in the *y*-direction. The slot effect occurs for the so-called quasi-TE polarization; the fundamental symmetric eigenmode is called quasi-TE_00_^12^. Similarly to the planar case, the property of increasing the repulsive forces, as the refractive index of the fluid medium increases, also happens to the antisymmetric eigenmodes of rectangular cross-section slot waveguides, as for instance, the quasi-TE_01_ mode. Figure 4 shows the FEM (Finite Element Method) simulations for the spatial distributions of the normalized major electric field component, *E*_*x*_(*x*, *y*), for a silicon rectangular slot waveguide with width *w* = 280 nm, height *h* = 240 nm, and gap g = 50 nm; field profiles are shown for the quasi-TE_00_ (left column) and quasi-TE_01_ (right column) polarizations, with structures immersed in Air shown in Fig. 4(a,b), H_2_0 (c,d), CS_2_ (e,f), and CH_3_I (g,h), respectively. Results show that, for the fluid with the lowest refractive index, the transverse component of the electric field at the quasi-TE_00_ polarization is highly confined in the gap region and, as the fluid refractive index increases, the field spreads out mostly over the structure. On the other hand, for the antisymmetric eigenmode quasi-TE_01_, the outer evanescent fields spread out mostly over the fluid as the fluid refractive index increases.

**Figure 5:**
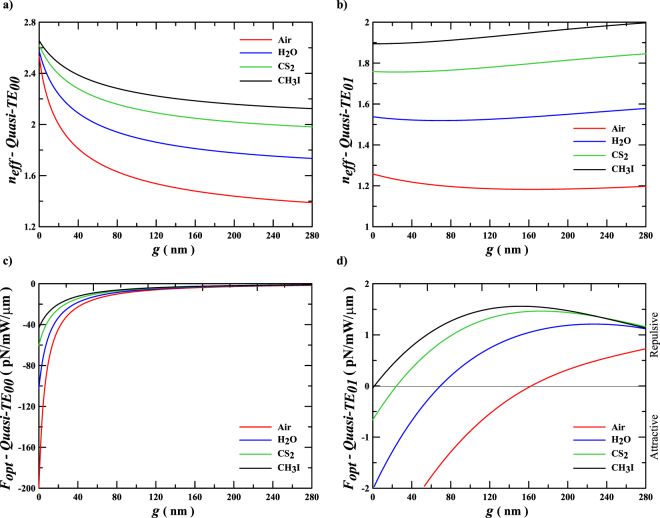
Effective indexes and optical forces of the dielectric rectangular slot waveguide. Effective indexes (**a,b**) and optical forces (**c,d**) of the silicon rectangular slot waveguide, with width *w* = 280 nm and high *h* = 240 nm at wavelength *λ*_0_ = 1550 nm, immersed in different dielectric media, as a function of the gap distance *g* for the fundamental symmetric mode quasi-TE_00_ (**a,c**) and the antisymmetric mode quasi-TE_01_ **(b**,**d)**.

The effective indexes and the optical forces for the symmetric and the antisymmetric eigenmodes at the quasi-TE polarization of the silicon rectangular slot waveguide are presented in Fig. 5. The effective indexes and the optical forces of the quasi-TE_00_ mode, shown in Fig. 5(a and c), respectively, present similar behaviors of the symmetric TM mode of the planar configuration, Fig. 3(a and c). The attractive optical force in the rectangular slot waveguide is −200 pN/mW/*µm* in Air and −43 pN/mW/*µm* in CH_3_I. On the other hand, Fig. 5(b) shows that the effective index of the quasi-TE_01_ mode in Air starts to decrease and then it increases again, as the gap increases, in contrast with what occurs in the planar structure. The point where this inversion of the effective index occurs is exactly where the cross-over gap happens, which switches the optical forces from attractive to repulsive as presented in^9^. However, Fig. 5(d) shows that, as the fluid medium refractive index increases the cross-over gap decreases, until its complete elimination in CH_3_I, turning the optical force solely repulsive for all gap intervals in rectangular waveguides. It is worth to mention that the cross-over gap, when it exists, represents an unstable point of operation, since it shows a behavior of attractive forces for narrower gaps and repulsive forces for wider gaps; such a condition leads to unique properties that may be exploited for unusual nano-optomechanical applications, such as bistability or memory, besides in manipulation and (bio) sensing, just to mention a few. This effect can also be used in conjunction with other approaches, in order to further increase the repulsive forces^21^. In addition, it is a feasible alternative to experimental demonstrations of the repulsive optical forces in very narrow gaps^22,23^. Furthermore, this effect may be applied to balance the attractive van der Waals and Casimir (dispersion) forces in nano-optomechanical structures, as these forces also decrease in liquids^20^. Therefore, this study also presents the theoretical foundations for tailoring the cross-over gap condition by appropriate design and choice of polarization eigenmode, gap distance and fluid cladding medium, in order to optimize the optomechanical structure for novel applications.

## Conclusions

In this report, we have described the behavior of optical forces in rectangular slot waveguides, as the refractive index of the fluid cladding medium varies. The revealed effects were underlined in stress tensor formalism version of the optical gradient forces. We demonstrated that both attractive and repulsive optical forces are feasible in realistic nano-optomechanical devices; we showed that, by increasing the fluid medium refraction index, it is possible to tailor the cross-over gap up to its complete elimination, which turns the optical force totally repulsive in all the gap intervals, for the antisymmetric modes. We have also proposed that the tailoring of the cross-over gap may be exploited for novel nano-optomechanical applications, such as bistability or memory. Finally, we highlight that the possibility of tailoring the attractive and the repulsive optical gradient forces, inside dielectric fluid media, may be applied to design active devices that enable optical-actuated mechanical motions inside nano-optofluidic devices.

## Methods

### Planar slot waveguides

The effective refractive indexes of the symmetric and antisymmetric modes presented in Fig. 3(a and b), respectively, of the planar slot waveguide, were obtained by solving their respective TM transcendental equations^11^, for each value of gap at a given fluid medium. Then, the derivatives of the effective indexes were numerically performed to obtain the optical forces shown in Fig. 3(c and d).

### Rectangular slot waveguides

The numerical simulations of the rectangular slot waveguide were done by using the finite element method with the RF Module of COMSOL Multiphysics® commercial software platform. The results presented in Figs 4(a–h) and 5(a and b) were simulated using a 2 × 2 *µm* ^2^ PEC (Perfect Electric Conductor) boundary condition with a tetrahedral adaptive mesh.

## Electronic supplementary material

Supplementary Information
