# Supplementary material for: Tailoring Optical Forces Behavior in Nano-optomechanical Devices Immersed in Fluid Media
Source: Sci Rep. 2017 Oct 30;7:14325. doi: 10.1038/s41598-017-14777-z (PMC5662605; doi:10.1038/s41598-017-14777-z)
Supplement: Supplementary file 1 — Supplementary Information [file 41598_2017_14777_MOESM1_ESM.pdf]

Tailoring Optical Forces Behavior in Nano-optomechanical Devices Immersed in Fluid Media

Janderson R. Rodrigues[1,2,*] and Vilson R. Almeida[1,2]

[1]Instituto Tecnológico de Aeronáutica, São José dos Campos - SP, 12228-900, Brasil
[2]Instituto de Estudos Avançados, São José dos Campos – SP, 12228-001, Brasil
*jrr@ita.br

Supplementary Information

The optical forces per unit area (length in z-direction and height in y-direction) in a planar slot waveguide can be formally obtained by calculating the Minkowski stress tensor in such a structure[11]; for the TM polarization modes, it is given by:

$$\frac{F_{optTM}(\mathrm{g})}{hL} = \pm\frac{\mu_0}{4}|A_{TM}|^2\left(1-\frac{n_{effTM}(\mathrm{g})^2}{n_L{}^2}\right) \tag{S1}$$

where μ_0 is the vacuum magnetic permeability, the "+" sign stands for the symmetric modes, whereas "–" stands for the antisymmetric modes, and n_{effTM} is the TM eigenmode effective index. The optical forces are proportional to the mode field intensities $|A_{TM}|^2$, which are obtained by applying the field spatial distributions in the time averaged Poynting vector[11]. For the symmetric TM modes, the optical power per unit length is given by:

$$\frac{P_{TMs}}{h} = \frac{n_{effTMs}|A_{TMs}|^2}{c_0\varepsilon_0}\left\{\frac{1}{n_L{}^2}\left[\frac{\mathrm{g}}{4}+\frac{\sinh(\gamma_S \mathrm{g})}{4\gamma_L}\right]+\frac{1}{n_H{}^2}\left[\frac{\cosh^2(\gamma_L\,\mathrm{g}/2)\sin(2\kappa_H w)}{4\kappa_H}+\right.\right.$$

$$+\frac{\cosh^2(\gamma_L\,\mathrm{g}/2)\,w}{2}+\frac{n_H{}^2}{n_L{}^2}\frac{\gamma_L}{\kappa_H{}^2}\cosh\left(\frac{\gamma_L \mathrm{g}}{2}\right)\sinh\left(\frac{\gamma_L \mathrm{g}}{2}\right)\sin^2(\kappa_H w)+$$

$$\left.-\frac{n_H{}^4}{n_L{}^4}\frac{\gamma_L{}^2}{4\kappa_H{}^3}\sinh^2\left(\frac{\gamma_L \mathrm{g}}{2}\right)\sin(2\kappa_H w)+\frac{n_H{}^4}{n_L{}^4}\frac{\gamma_L{}^2}{2\kappa_H{}^2}\sinh^2\left(\frac{\gamma_L \mathrm{g}}{2}\right)w\right]+$$

$$\left.+\frac{1}{n_L{}^2 2\gamma_L}\left[\cosh\left(\frac{\gamma_L \mathrm{g}}{2}\right)\cos(\kappa_H w)+\frac{n_H{}^2}{n_L{}^2}\frac{\gamma_L}{\kappa_H}\sinh\left(\frac{\gamma_L \mathrm{g}}{2}\right)\sin(\kappa_H w)\right]^2\right\} \tag{S2}$$

and for the antisymmetric TM mode, it is given by:

$$\frac{P_{TMa}}{h} = \frac{n_{effTMa}(\mathrm{g})|A_{TMa}|^2}{c_0\varepsilon_0}\left\{\frac{1}{n_L{}^2}\left[\frac{\sinh(\gamma_L \mathrm{g})}{4\gamma_L}-\frac{\mathrm{g}}{4}\right]+\frac{1}{n_H{}^2}\left[\frac{\sinh^2(\gamma_L\,\mathrm{g}/2)\sin(2\kappa_H w)}{4\kappa_H}+\right.\right.$$

$$+\frac{\sinh^2(\gamma_L\,\mathrm{g}/2)\,w}{2}+\frac{n_H{}^2}{n_L{}^2}\frac{\gamma_L}{\kappa_H{}^2}\sinh\left(\frac{\gamma_L \mathrm{g}}{2}\right)\cosh\left(\frac{\gamma_L \mathrm{g}}{2}\right)\sin^2(\kappa_H w)+$$

$$\left.-\frac{n_H{}^4}{n_L{}^4}\frac{\gamma_L{}^2}{4\kappa_H{}^3}\cosh^2\left(\frac{\gamma_L \mathrm{g}}{2}\right)\sin(2\kappa_H w)+\frac{n_H{}^4}{n_L{}^4}\frac{\gamma_L{}^2}{2\kappa_H{}^2}\cosh^2\left(\frac{\gamma_L \mathrm{g}}{2}\right)w\right]+$$

$$+\frac{1}{2\gamma_L n_L{}^2}\left[\sinh\left(\frac{\gamma_L g}{2}\right)\cos[\kappa_H w]+\frac{n_H{}^2}{n_L{}^2}\frac{\gamma_L}{\kappa_H}\cosh\left(\frac{\gamma_L g}{2}\right)\sin(\kappa_H w)\right]^2\Bigg\} \quad \text{(S3)}$$

where ε_0 and c_0 are the vacuum electric permittivity and the speed of the light, respectively; κ_H and γ_L are the transversal wavenumber and the field decay coefficient, respectively.

In the limit of very small gaps (g → 0), the optical power per unit length of the symmetric TM modes converges to:

$$\frac{P_{TMs}}{h}=\frac{n_{effTMs}|A_{TMs}|^2}{c_0\varepsilon_0}\left[\frac{\sin(2\kappa_H w)}{4\kappa_H n_H{}^2}+\frac{w}{2n_H{}^2}+\frac{\cos^2(\kappa_H w)}{2n_L{}^2\gamma_L}\right] \quad \text{(S4)}$$

which leads to

$$|A_{TMs}|^2=\frac{(P_{TMs}/h)c_0\varepsilon_0}{n_{effTMs}}\left[\frac{\sin(2\kappa_H w)}{4\kappa_H n_H{}^2}+\frac{w}{2n_H{}^2}+\frac{\cos^2(\kappa_H w)}{2n_L{}^2\gamma_L}\right]^{-1} \quad \text{(S5)}$$

However, the optical power per unit length of the antisymmetric TM modes, in the limit of a very narrow gap, tends to:

$$\frac{P_{TMa}}{h}=\frac{n_{effTMa}|A_{TMa}|^2}{c_0\varepsilon_0}\frac{1}{n_L{}^4}\left[-\frac{n_H{}^2\gamma_L{}^2}{4\kappa_H{}^3}\sin(2\kappa_H w)+\frac{n_H{}^2\gamma_L{}^2}{2\kappa_H{}^2}w+\frac{n_H{}^4}{2n_L{}^2}\frac{\gamma_L}{\kappa_H{}^2}\sin^2(\kappa_H w)\right] \quad \text{(S6)}$$

which yields the following relation,

$$|A_{TMa}|^2=\frac{(P_{TMa}/h)c_0\varepsilon_0}{n_{effTMa}}n_L{}^4\left[-\frac{n_H{}^2\gamma_L{}^2}{4\kappa_H{}^3}\sin(2\kappa_H w)+\frac{n_H{}^2\gamma_L{}^2}{2\kappa_H{}^2}w+\frac{n_H{}^4}{2n_L{}^2}\frac{\gamma_L}{\kappa_H{}^2}\sin^2(\kappa_H w)\right]^{-1} \quad \text{(S7)}$$

By analyzing Eqs. (S5) and (S7), it is possible to show that $|A_{TMa}|^2$ for the antisymmetric TM modes is, under certain conditions, proportional to the fourth power of the fluid medium index, i.e., $|A_{TMa}|^2 \propto n_L{}^4$, which does not happen to the symmetric modes; therefore, by substituting Eq. (S7) in the antisymmetric version of Eq. (S1), we notice that the optical forces of these modes have a high sensibility to the fluid medium refractive index. However, it is worthy to notice that, even though the squared field amplitude keeps increasing as the fluid medium refractive index increases, the term in parentheses of Eq. (S1) tends to decrease, limiting this effect.
